# Statistics of Midwifery

**Published:** 1844-05

**Authors:** 


*Statistics of Midwifery.*—The relation between the time of the last menstruation and the birth of the child is an interesting one. It was noted in 259 cases, as follows:

| | | | | | | | |
|---|---|---|---|---|---|---|---|
| In 1 | case | menstruation | occurred | about | 5½ | months | before delivery. |
| 4 | cases | " | " | " | 6 | " | " " |
| 1 | case | " | " | " | 6½ | " | " " |
| 9 | cases | " | " | " | 7 | " | " " |
| 2 | " | " | " | " | 7½ | " | " " |
| 24 | " | " | " | " | 8 | " | " " |
| 24 | " | " | " | " | 8½ | " | " " |
| 138 | " | " | " | " | 9 | " | " " |
| 26 | " | " | " | " | 9½ | " | " " |
| 18 | " | " | " | " | 10 | " | " " |
| 11 | " | " | " | " | 11 | " and over | " |
| 1 | case | " | " | at irregular intervals throughout gestation, the appearance of the catamenia being preceded by violent neuralgic pains. | | | |

The points of interest in this table are—1st, the comparatively small number of cases where the intervening time has been less than 8 months; 18 in 259 cases, or in the proportion of 1 to 15; of these 18 cases, labor came on in nine of them at 7 months. This large proportion, taken in connection with the results, would seem to indicate a disposition of nature to expel the child at the seventh month of the utero-gestation. The question would arise, also, whether the presentation of the child had anything to do with this. The case at 5½ months was a woman who dated her pregnancy nine months before her delivery, but who continued to menstruate regularly until this short period before her accouchement. Child healthy and weighed 6 pounds 11 ounces.

Total duration of labor. It was noted in 526 cases and lasted

| | | | | | |
|---|---|---|---|---|---|
| 3 hours and under, | in 77 | cases. | From 24 to 36 hours | in 35 | cases. |
| From 3 to 6 hours | in 73 | " | 36 to 48 " | in 13 | " |
| 6 to 12 " | 162 | " | 48 to 72 " | in 8 | " |
| 12 to 18 " | 95 | " | 4 days | in 1 | " |
| 18 to 24 " | 61 | " | 8½ " | in 1 | " |

The proportion of those under six hours to the whole number is as 1 to 3:48 or 28 per cent.

Of *nineteen* still-born children where the superior extremity of the fœtal ellipse presented, in *eight* of them no cause was ascertained, nor did there anything occur during the delivery that might lead to such a result. *Two* had the cord tightly around the neck at birth, and the face livid. In both the cord was loosened as soon as possible, and efforts made to resuscitate without effect. *Two* breathed after birth, but soon died, one in half an hour, and the other in five hours. *Three* were from mothers who had convulsions during labor. *One* was still-born after a labor of fifty-eight hours in a woman with venereal warts. *One* was the first of twins and "had been dead some time." *One* had its position changed from the 4th vertex to the 2d vertex during the progress of the labor, and *had the cord around its neck.* It lived half an hour and died. (Can the fact of the cord being around the neck endanger the child during such a change of position?) In *one* case ergot was given during the second stage, and repeated with effect eight and a half hours before the delivery.—*Am. Jour. Med. Sci.*, April.

